# Supplementary material for: Protein Malnutrition Facilitates Intestinal Colonization with Highly Resistant Klebsiella pneumoniae
Source: bioRxiv. 2025 Jul 21:2025.07.21.665917. Preprint. [Version 1] doi: 10.1101/2025.07.21.665917 (PMC12330750; doi:10.1101/2025.07.21.665917)
Supplement: Supplement 1 [file NIHPP2025.07.21.665917v1-supplement-1.pdf]

bioRxiv preprint doi: https://doi.org/10.1101/2025.07.21.665917; this version posted July 21, 2025. The copyright holder for this preprint (which was not certified by peer review) is the author/funder, who has granted bioRxiv a license to display the preprint in perpetuity. It is made available under aCC-BY 4.0 International license.

Supplemental Material:

Supplemental Figure 1

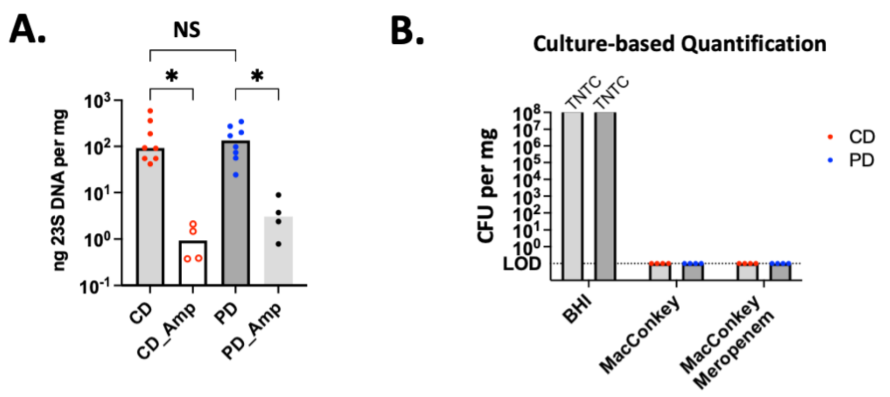

Figure S1: Quantification of Bacteria in Feces and Ceca of Mice fed CD or PD
(A.) Fecal specimens were collected from CD- and PD-fed mice, with or without ampicillin in their water for the previous 7 days. qPCR detection of 23S rRNA content was used for total bacterial burden quantification. Median concentration depicted, N=4-8 per group. Mann-Whitney U-test with 10% FDR performed, *= p<0.05, NS= No significance. (B.) Burden of aerobic bacteria expressed as log CFU per mg of cecal content growing on BHI, MacConkey, and MacConkey + 0.125 ug/ml meropenem agar in mice initiated on respective diets 7 days prior to sacrifice. Median concentration depicted. N = 4 per group. TNTC = too numerous to count.

bioRxiv preprint doi: https://doi.org/10.1101/2025.07.21.665917; this version posted July 21, 2025. The copyright holder for this preprint (which was not certified by peer review) is the author/funder, who has granted bioRxiv a license to display the preprint in perpetuity. It is made available under aCC-BY 4.0 International license.

Supplemental Figure 2

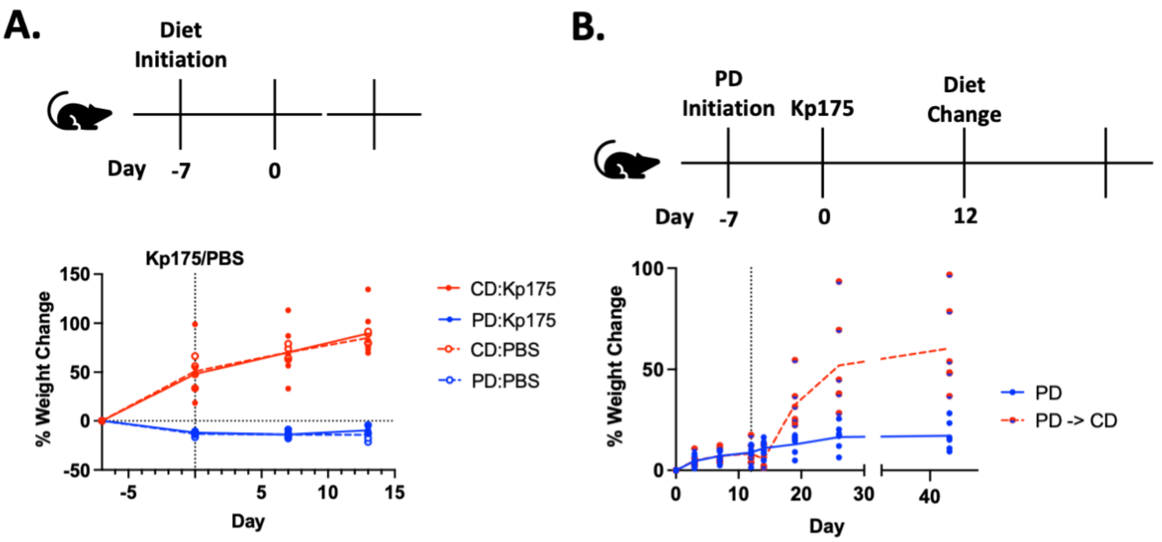

Figure S2: Dietary effect on mouse weight
(A.) Mice were initiated on CD or PD 7 days prior to oral gavage with 10^6 CFU Kp175 or PBS, weight change expressed relative to day of diet initiation. N=4 per group. (B.) Mice were initiated on PD 7 days prior to oral gavage with 10^6 CFU Kp175 or PBS, and in half of the mice diet switched to CD on day 12. Weight change expressed relative to day of inoculation. Mean weight change depicted, N=6 per group.

Supplemental Figure 3

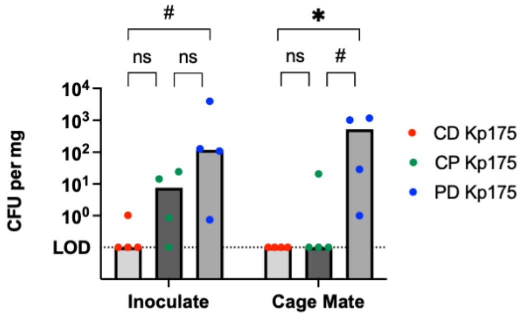

Figure S3: Peak Kp175 Burden and Transmission in Mice Fed CD, PD, or CP
3-4 week old C57BL/6 mice placed on respective diets 7 days prior to inoculation with 10^6 CFU Kp175 or PBS, with one Kp175 inoculated mouse and one PBS control cage-mate placed together in a single cage on day 2. Day 10 Kp175 burden expressed as log CFU per mg fecal specimen. Median burden depicted, N = 4 per group. Mann-Whitney U-test with 10% FDR performed, *= $p<0.05$, #= $p<0.1$, ns= no significance

bioRxiv preprint doi: https://doi.org/10.1101/2025.07.21.665917; this version posted July 21, 2025. The copyright holder for this preprint (which was not certified by peer review) is the author/funder, who has granted bioRxiv a license to display the preprint in perpetuity. It is made available under aCC-BY 4.0 International license.

Supplemental Figure 4

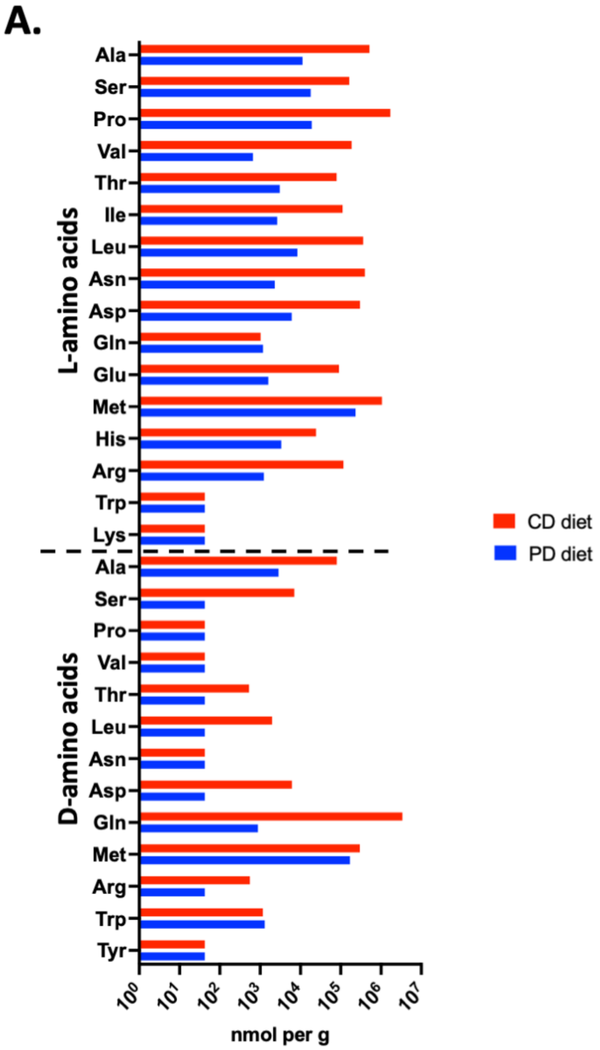

bioRxiv preprint doi: https://doi.org/10.1101/2025.07.21.665917; this version posted July 21, 2025. The copyright holder for this preprint (which was not certified by peer review) is the author/funder, who has granted bioRxiv a license to display the preprint in perpetuity. It is made available under aCC-BY 4.0 International license.

Supplemental Figure 4

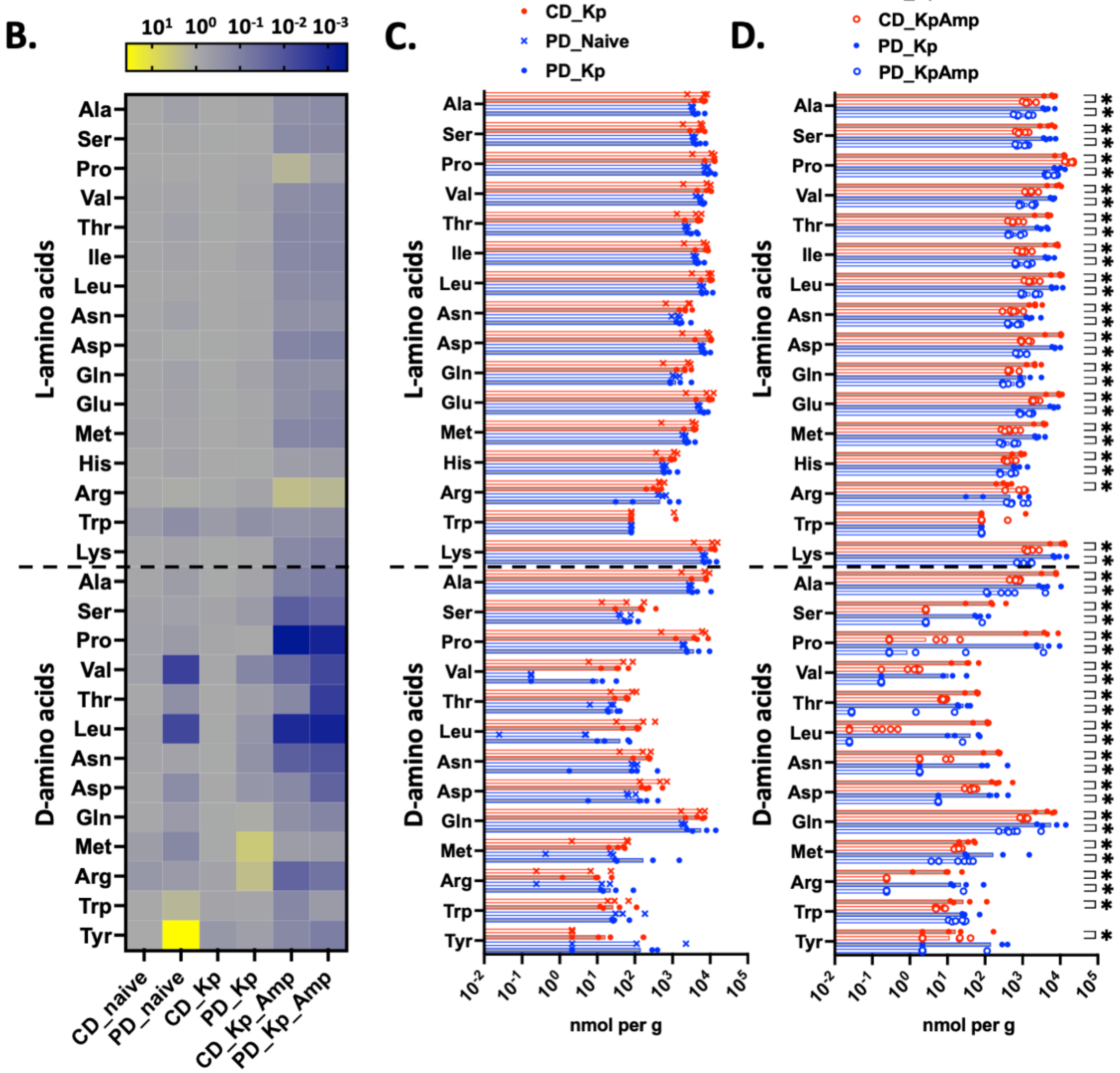

Figure S4: *Amino Acid Content in Ceca of CD- and PD-fed Mice and in Mouse Food*
(A.) Concentration of individual free L- and D-amino acids in CD or PD food. (B.) Heat map of individual free L- and D-amino acids in cecal content of mice placed on respective diets 7 days prior to inoculation with 10^6 CFU Kp175 (or naïve without inoculation) and terminated on day 14 post-inoculation or treated with Ampicillin in the drinking water (1 mg/ml) at day 35 post-inoculation and terminated at day 42. Expressed as fold change relative to average concentration in cecal contents of colonized CD-fed mice without antibiotic treatment. Median concentration depicted, N=3-6 per group. (C.) Concentration of individual free L- and D-amino acids in cecal content of mice placed on respective diets 7 days prior to inoculation with 10^6 CFU Kp175 or left without inoculation (naïve) and terminated on day 14 post-inoculation. Median concentration depicted, N=3-4 per group. Mann-Whitney U-test with 10% FDR performed. (D.) Concentration of individual free L- and D-amino acids in cecal content of mice placed on respective diets 7 days prior to inoculation with 10^6 CFU Kp175 treated with Ampicillin in the

bioRxiv preprint doi: https://doi.org/10.1101/2025.07.21.665917; this version posted July 21, 2025. The copyright holder for this preprint (which was not certified by peer review) is the author/funder, who has granted bioRxiv a license to display the preprint in perpetuity. It is made available under aCC-BY 4.0 International license.

drinking water (1 mg/ml) at day 35 post-inoculation and terminated at day 42. Median concentration depicted, N=4-6 per group, Mann-Whitney U-test with 10% FDR performed, *= p<0.05 by Mann-Whitney U-test.

Supplemental Figure 5

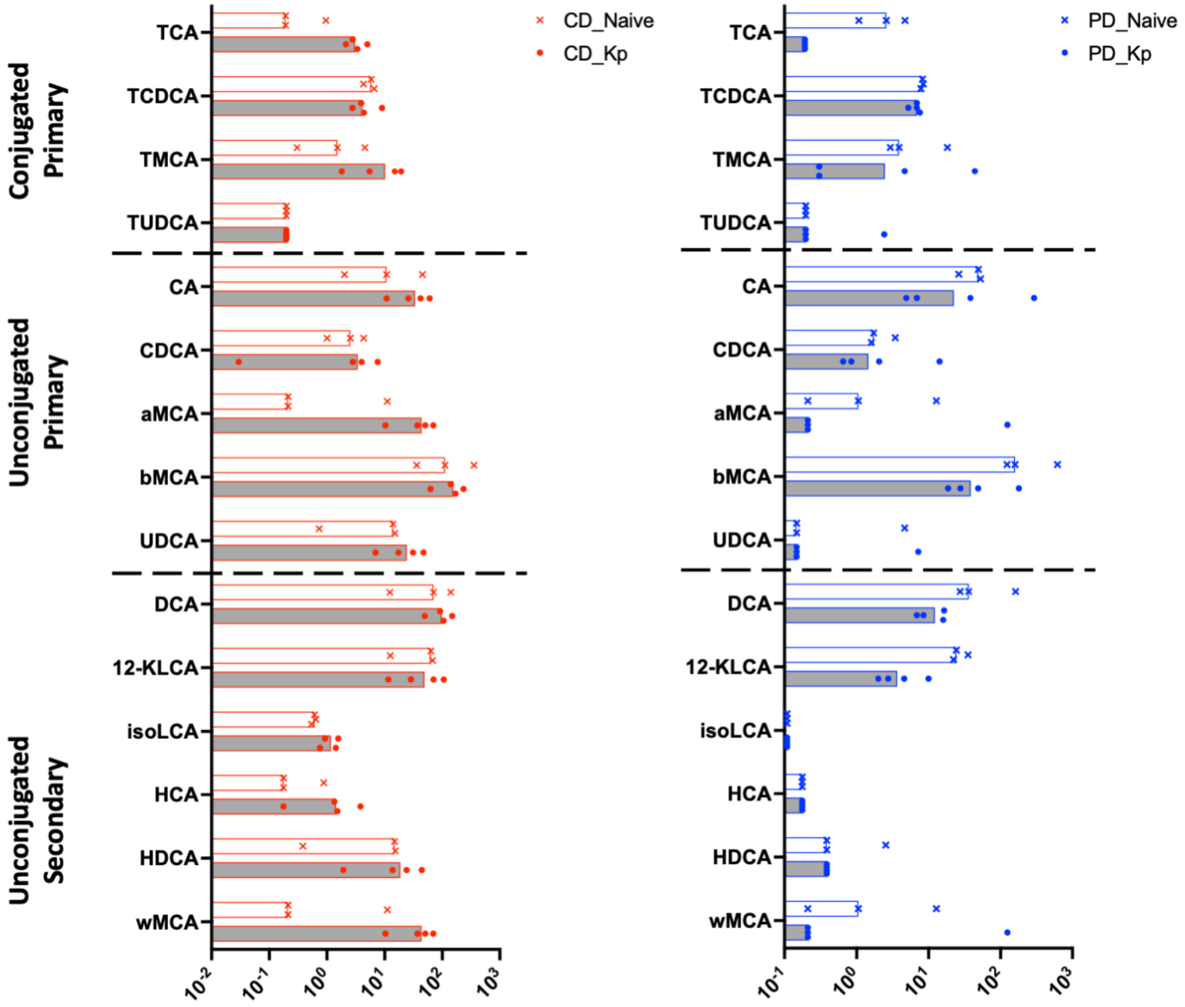

Figure S5: Bile Acid Content in Ceca of Mice With or Without Kp175 Colonization
Concentration of individual L- and D-amino acids in cecal content of mice placed on respective diets 7 days prior to oral gavage with 10^6 CFU Kp175 or left without inoculation (naïve) and terminated on day 14 post-inoculation. Median concentration depicted, N=3-4 per group. Mann-Whitney U-test with 10% FDR performed. *TCA= Taurocholic Acid, TCDCA= Taurochenodeoxycholic Acid, TMCA= Tauromuricholic Acid, TUDCA= Tauroursodeoxycholic Acid, CA= Cholic Acid, CDCA= Chenodeoxycholic Acid, aMCA= Alpha Muricholic Acid, bMCA= Beta Muricholic Acid, UDCA= Ursodeoxycholic Acid, DCA= Deoxycholic Acid, 12-KLCA= 12-Ketolithocholic Acid, isoLCA= Isolithocholic Acid, HCA= Hyocholic Acid, HDCA= Hyodeoxycholic Acid, wMCA= Omega Muricholic Acid.*

bioRxiv preprint doi: https://doi.org/10.1101/2025.07.21.665917; this version posted July 21, 2025. The copyright holder for this preprint (which was not certified by peer review) is the author/funder, who has granted bioRxiv a license to display the preprint in perpetuity. It is made available under aCC-BY 4.0 International license.

Table S1:

Isolate ID	Sequence Type	CR/ESBL Genes	Culture Source	Clinical History
Kp175	ST307	OXA-48 CTXM-15	Rectal Swab	Middle-aged Male, positive culture 114d after index, household transmission documented
ARLG-4605	ST11	KPC-2	Sputum	Elderly Female, subsequently expired
ARLG-4404	ST378	KPC-2 CTXM15	Urine	Middle-aged Female, documented urinary tract infection

Table S1: Carbapenem Resistant Klebsiella pneumoniae Isolates
Sequence type, antimicrobial resistance genes, and clinical features of isolates used in study.

Table S2:

	R^2	Pearson r	P value
TCA	0.6301	0.726	0.0003
TCDCA	0.01925	-0.4632	0.0397
TMCA	0.3116	0.6423	0.0023
TUDCA	0.4946	0.676	0.0011
**Total Conjugated**	**0.6185**	0.7534	**0.0001**
CA	0.0487	-0.6762	0.0011
CDCA	0.1598	-0.688	0.0008
aMCA	0.2434	-0.6561	0.0017
bMCA	0.6264	-0.7142	0.0004
UDCA	0.5751	-0.6858	0.0008
**Total Unconjugated**	**0.3518**	-0.7108	**0.0004**
DCA	0.7448	-0.8605	<0.0001
12-KLCA	0.558	-0.8707	<0.0001
isoLCA	0.7437	-0.6862	0.0008
HCA	0.4066	-0.5418	0.0136
HDCA	0.4693	-0.684	0.0009
wMCA	0.2434	-0.6561	0.0017
**Total Secondary**	**0.6606**	-0.8503	**<0.0001**

bioRxiv preprint doi: https://doi.org/10.1101/2025.07.21.665917; this version posted July 21, 2025. The copyright holder for this preprint (which was not certified by peer review) is the author/funder, who has granted bioRxiv a license to display the preprint in perpetuity. It is made available under aCC-BY 4.0 International license.

Table S2: Bile Acid Content in Ceca of Colonized Mice With or Without Ampicillin Treatment
Primary and secondary bile acid content and Kp175 burden compared from ceca of mice placed on respective diets 7 days prior to inoculation with 10^6 CFU and terminated on day 14 post-inoculation or treated with Ampicillin in the drinking water (1 mg/ml) at day 35 post-inoculation and sacrificed at day 42. R^2 calculated with least squares regression with X as logarithmic variable and Y as linear variable and r and p calculated using Spearman correlation method. *TCA= Taurocholic Acid, TCDCA= Taurochenodeoxycholic Acid, TMCA= Tauromuricholic Acid, TUDCA= Tauroursodeoxycholic Acid, CA= Cholic Acid, CDCA= Chenodeoxycholic Acid, aMCA= Alpha Muricholic Acid, bMCA= Beta Muricholic Acid, UDCA= Ursodeoxycholic Acid, DCA= Deoxycholic Acid, 12-KLCA= 12-Ketolithocholic Acid, isoLCA= Isolithocholic Acid, HCA= Hyocholic Acid, HDCA= Hyodeoxycholic Acid, wMCA= Omega Muricholic Acid.*
